# 3D-printed pulsator to enhance mass transfer in electrochemical reactors

**DOI:** 10.1016/j.ohx.2025.e00645

**Published:** 2025-03-27

**Authors:** Kavin Teenakul, Luis Fernando Arenas, Jonas Hereijgers

**Affiliations:** Research Group Applied Electrochemistry & Catalysis, University of Antwerp, Universiteitsplein 1, 2610 Wilrijk, Belgium

**Keywords:** Diaphragm pulsator, Electrochemical reactor, Mass transport enhancement, Sinusoidal flow profile, Pulsating flow

## Abstract

This study presents a cost-effective diaphragm pulsator, constructed for approximately €500, designed to enhance mass transport in laboratory electrochemical reactors. The pulsator allows accurate control of pulsation frequency between 1 Hz and 6 Hz and displacement volume, with simple programmability using an Arduino microcontroller. The design features multiple chambers that effectively isolate corrosive liquids from the mechanical components, ensuring durability and extended operational life. The pulsator’s 3D-printed components can be customized with different materials to suit various applications. Engineered to generate a pulsating flow profile that closely resembles a sinusoidal wave, video tracking analysis confirmed the sinusoidal nature of the flow, demonstrating consistent flow profile generation with adjustable frequency and amplitude. The maximum volume displacement achieved was 11.9 mL, which was reduced to 2.0 mL when the electrochemical cell was connected. Limiting current experiments with a ferri/ferrocyanide electrolyte showed that the mass transport coefficient of a typical cell increased from 2.3 × 10^−3^ cm/s under constant flow to 4.5 × 10^−3^ cm/s under pulsating conditions. These findings validate that the adjustable, Arduino-programmable sinusoidal pulsation generated by the diaphragm pulsator offers a practical and customizable method for enhancing mass transport in small-scale electrochemical reactors.

## Graphical abstract

**Figure:**
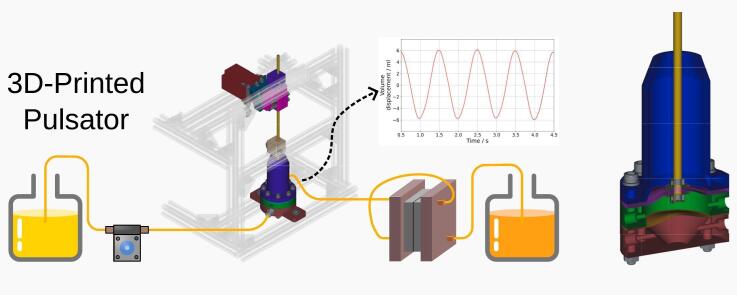

## 1 Specifications table

**Table:** 

Hardware name	3D-Printed Pulsator for Electrochemical Reactors
Subject area	− Engineering and materials science
Hardware type	− Mechanical engineering and materials science − Electrochemical engineering
Closest commercial analog	Prominent Sigma/1 Basic Type (S1Ba) 07,065 PVT
Open-source license	CC-BY-4.0
Cost of hardware	€500
Source file repository	https://doi.org/10.5281/zenodo.14221857

## 2 Hardware in context

In electrochemical reactors, an adequate mass transport of electroactive species to the electrode surface is essential for sustaining fast reaction rates and overall high energy efficiency [1], [2]. One method to improve mass transport is by generating a pulsating flow of electrolyte, which creates periodic variations in local fluid velocity together with eddies, mixing and turbulence [3], [4], [5], [6], [7]. Diaphragm pulsators can play a crucial role in this process by generating forward and backward fluid flow, resulting in the desired pulsating effect. However, many electrolytes used in electrochemical applications are caustic (e.g. alkaline water electrolysis [8]), acidic (e.g. vanadium flow battery [9]) or air-sensitive (e.g. organic flow batteries [10]), requiring devices made from materials that can withstand these harsh conditions and/or ensure an inert atmosphere. This makes material compatibility a significant concern that low-cost commercial pumps may not adequately address.

Commercial diaphragm pumps can be used to produce a pulsating flow by removing their check valves [5], [7]. Devices with suitable materials, adjustable pulse volume and frequency controls exist, but they are expensive. Moreover, these pumps often have a limited range of settings for pulse volume and frequency, as they are not intended to produce well-defined pulsating flows. If researchers require specific pulse parameters outside of these ranges, they might need to purchase or modify entirely new pumps, leading to additional costs and logistical challenges. An example of a commercial diaphragm pump suitable for corrosive electrolyte is Prominent Sigma/1 Basic Type (S1Ba) 07065 PVT [11], which can create a maximum pulse volume of 5.2 mL and a maximum frequency of 4.1 Hz and has a cost of approximately €2000.

There is a need for a highly customizable, airtight, and watertight diaphragm pulsator that allows for precise control over amplitude, frequency, and automation while being compatible with a variety of electrolytes. Such a device would enable researchers to tailor the pulsating flow to their specific experimental requirements, facilitating more effective studies of mass transport effects in laboratory-scale electrochemical systems. The advent of fused deposition modelling (FDM) and stereolithography (SLA) 3D printing has made fabrication of custom parts and specialized components cheaper and faster without the need for outsourcing [12]. This technology allows for the selection of appropriate materials that are compatible with specific electrolytes, addressing the issue of material compatibility directly. Additionally, open-source electronic platforms like Arduino provide low-cost, programmable microcontrollers with user-friendly syntax, making automation accessible even to researchers with minimal programming or electronics background. Arduino has been widely adopted in research environments due to its versatility and ease of use [13]. Furthermore, its capability can be extended with accessories for additional control of an experiment, for example, to respond to a change in the electrochemical cell conditions or to detect an electrolyte leakage and stop the pulsator and the pump.

Our work addresses the challenges of integrating pulsating flow into electrochemical reactors by providing a device that meets stringent material compatibility and air-sensitivity requirements of electrolytes. The adjustable flow frequency and volume, along with programmable automation, offer significant benefits for future research in this area. Following a low-cost, free-access approach, the physical design of the pulsator was carried out with FreeCAD version 1.1.0dev [14], an open-source CAD software, enabling precise and customizable designs without additional software costs. Moreover, the flow profiles generated by the diaphragm pulsator were characterized using Tracker version 6.2.0 [15], an open-source video tracking and modelling software, which allowed for detailed analysis of the pulsating flow. Open-source tools and affordable technologies are leveraged to provide a customizable and accessible diaphragm pulsator for studying mass transport effects in electrochemical reactors. Furthermore, the device offers adaptability for other applications requiring precise pulsating fluid flow control.

## 3 Hardware description

The do-it-yourself (DIY) diaphragm pulsator was designed with customizable materials, adjustable flow parameters, and an airtight electrolyte chamber to address the relevant research requirements. An Arduino UNO R3 was used to generate electrical signals to a TB6600 stepper motor driver, which powers a NEMA23-05 motor. Additionally, Arduino UNO R3 can be used to control the stepper motor based on time and measured voltage on the analogue input pins. The diaphragm pulsator was driven by a periodic linear motion generated from the rotary motion of the NEMA23-05 stepper motor using a 3D-printed Scotch yoke mechanism. Some commercially available diaphragm pumps employ solenoid actuators that generate linear motion akin to a rectangular wave.

**Fig. 1:**
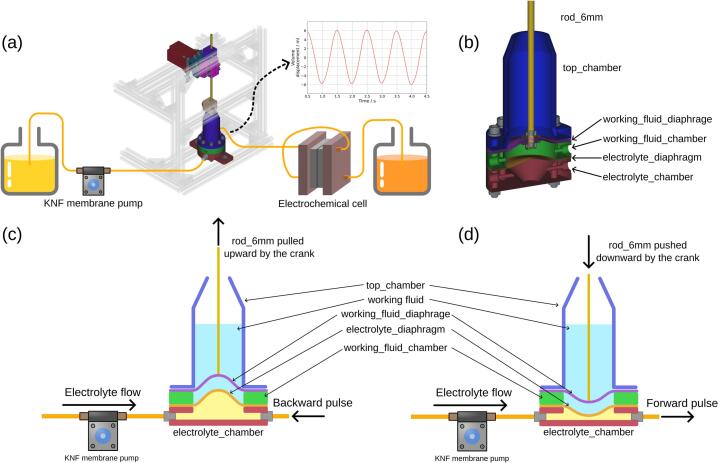
(a) Overview of the electrochemical setup for the limiting current experiment. The diaphragm pulsator generates a sinusoidal electrolyte flow into the electrochemical cell. (b) Cross-sectional view of the pulsator, illustrating that the diaphragm pulsator is composed of three chambers: the top_chamber, working_fluid_chamber, and electrolyte_chamber. (c) Schematic of the pulsator's working principle: as the crank pulls the rod_6mm upward, electrolyte_diaphragm generates a backward pulse flow that draws electrolyte toward the KNF membrane pump. (d) Conversely, when the crank pushes the rod_6mm downward, electrolyte_diaphragm creates a forward pulse flow that expels electrolyte away from the pump.

The overview of the electrochemical experimental setup is shown in Fig. 1 (a), where the pristine electrolyte was pumped into the electrochemical flow cell using a membrane pump, while pulsation was generated by the diaphragm pulsator. The diaphragm pulsator consisted of three chambers, as illustrated in Fig. 1 (b): 1) electrolyte chamber, 2) working fluid chamber, and 3) working fluid containment chamber. The first diaphragm, selected for chemical compatibility with the electrolyte (see below), was placed between the electrolyte chamber and the working fluid chamber. A second diaphragm, linked by a sliding yoke to a connecting rod, was placed between the working fluid chamber and the working fluid containment chamber. The purpose of incorporating the working fluid and the working fluid chamber was to transfer mechanical force from the connecting rod to the first diaphragm without needing to make a hole through the diaphragm. Directly connecting the stainless-steel rod to the diaphragm would require complex sealing to resist corrosion when in contact with the electrolyte or the use of adhesives, which can fail during extended operation. The working fluid containment chamber was used to contain any leakage of working fluid and to prevent air from entering the working fluid chamber by being partially filled.

Since no holes were made in the first diaphragm that faced the electrolyte, hermeticity and airtightness was ensured, and the material compatibility of the connecting rod needed to be considered only for the working fluid, which in this design was water. Other working fluids, such as oil, are also possible. The diaphragm facing the electrolyte was made by cutting a commercially available elastic material; in this design, a fluoroelastomer rubber (FKM) sheet was used due to its chemical resistant properties. The diaphragm bolted to the connecting rod was made from ethylene propylene diene monomer (EPDM) rubber sheet due to less stringent requirements for chemical compatibility [16]. The inlet and outlet ports on the electrolyte chamber allowed the electrolyte to flow through, while the inlet and outlet ports on the casing of the working fluid chamber were for filling the chamber with working fluid. Both filling ports were closed during operation of the pulsator.

The diaphragm pulsator provides a tuneable device for improving mass transport to the electrode surface in laboratory electrochemical reactors. The ability to control pulse volume, frequency, and programmable automation allows for optimized studies of mass transfer in various electrochemical technologies. Although a peristaltic pump does generate a pulsatile flow, the pulse frequency and volume are limited and cannot be independently controlled, the pulse frequency depends on the number of rollers and the rotational speed, while the pulse volume is determined by the tube diameter.

Diaphragm pulsator main features are:

• The use of customizable and chemically resistant materials ensures that the pulsator can handle corrosive electrolytes, such as acidic solutions or caustic solutions.
• Arduino microcontrollers provide programmable control over frequency and automation. Extended features such as cell voltage measurement and leak detection can be added.
• Pulse volume can be customized by printing larger electrolyte chamber and increase crank radius to provide large pulse in one stroke compared to a more expensive commercial diaphragm pump. Depending on the requirement, electric motor can be swapped to provide suitable speed and torque.

Finally, standard aluminium profiles provided structural support for the motor and the pulsator in which the dimensions of the enclosure set spaces for ease of service and maintenance as well as the possibility of adding more pulsator components plus sensors and displays.

### 3.1 Design files

The design file for 3D-Printed Pulsator for Electrochemical Reactors was designed with FreeCAD, a free and open-source software and is available as FreeCAD Standard file format(.FCStd). The parts name inside the ‘*3DPrint Pulsator for Electrochemistry_Build_Instruction.FCStd’ file* are named accordingly to match the name in the build instruction. The electrical connections for stepper motor to the Arduino are available with the article and the source code are available in constant_speed.ino, increase_speed_step.ino, and increase_speed_step_loop.ino.

## 4 Design files summary

**Table:** 

Design file name	File type	Open-source license	Location of the file
3DPrint Pulsator for Electrochemistry_Build_Instruction.FCStd	FreeCAD CAD file	CC-BY-4.0	https://doi.org/10.5281/zenodo.14221857
constant_speed.ino	Arduino sketch file	GPL-3.0-or-later	https://doi.org/10.5281/zenodo.14221857
increase_speed_step.ino	Arduino sketch file	GPL-3.0-or-later	https://doi.org/10.5281/zenodo.14221857
increase_speed_step_loop.ino	Arduino sketch file	GPL-3.0-or-later	https://doi.org/10.5281/zenodo.14221857

## 5 Bill of materials

**Table:** 

Designator	Component	Number	Cost per unit	Total cost	Source of materials	Material type
NEMA-23–05	Joy-IT NEMA-23 stepper motor	1	€ 31.99	€ 31.99	conrad.be 2355879–62	Metal
Arduino	Arduino A000066 Board UNO Rev3 DIL Core ATMega328	1	€ 26.04	€ 26.04	conrad.be 1275279 – 62	Composite
48 V power supply	Phihong PPL65W-480 Bench PSU (fixed voltage) 48 V DC 1.36 A 65.3 W	1	€ 29.99	€ 29.99	conrad.be 2355755 – 62	Composite
5 V power supply	Mean Well GST36E05-P1J Mains PSU (fixed voltage) 5 V DC 4300 mA 21.5 W	1	€ 17.99	€ 17.99	conrad.be 1439198 – 62	Composite
ENA switch	TRU COMPONENTS TC-9218544 Toggle switch 250 V AC 2 A 1 x On/On latch	1	€ 0.84	€ 0.84	conrad.be 2304636 – 62	Composite
linear_ball_bearing	Reely Linear ball bearing Inside diameter: 6 mm Outside diameter: 12 mm	2	€ 13.99	€ 27.98	conrad.be 221974 – 62	Composite
ball_bearing	Reely Radial ball bearing Chrome steel Inside diameter: 6 mm Outside diameter: 19 mm Rotational speed (max.): 40,000 U/m	1	€ 2.79	€ 2.79	conrad.be 214469 – 62	Metal
rod_6mm	RS PRO Stainless Steel Rod 6 mm Diameter, 1 m length	1	€ 9.76	€ 9.76	rs-online.com 682–826	Metal
grease	CRC Multi-Purpose Grease 100 mL	1	€ 6.99	€ 6.99	conrad.com 828658 – 62	Organic
stepper motor driver	Joy-it SBC-MD-TB6600 Stepper motor drive	1	€ 23.99	€ 23.99	conrad.com 2999126 – 62	Composite
tubing clamp	Tubing clamps, PVDF, LaboPlast	4	€ 4.42	€ 17.68	vwr.com 229–0609	Composite
PVC tube	Nalgen Metric Non-Phthalate PVC Tubing, ID 4 mm, OD 6 mm (Estimate cost for 50 cm per piece)	4	€ 3.41	€ 13.63	vwr.com NALG8703-0406	Polymer
G-clamp	WorkPro W032019WE 100 mm C-Clamp	1	€ 7.99	€ 7.99	conrad.com 2465027 – 62	Metal
aluminium_bracket_motor	Aluminium plate thickness: 10 mm (estimated cost)	2	€ 2.00	€ 4.00	vigotec.be	Metal
T-Slot nut M6	RS PRO M6 T-Slot Nut Connecting Component, Strut Profile 30 mm, Groove Size 6 mm	38	€ 1.26	€ 47.73	rs-online.com 767-5531	Metal
M6x80-Bolt-DIN933	DIN 933 M6 x 80 stainless steel bolt	2	€ 0.40	€ 0.80	conrad.com 1061895 – 62	Metal
M6x50-Bolt-DIN912	DIN 912 M6 x 50 stainless steel bolt	2	€ 0.31	€ 0.62	conrad.com 1202727 – 62	Metal
M6x60-Bolt-DIN912	DIN 912 M6 x 60 stainless steel bolt	8	€ 0.40	€ 3.20	conrad.com 1202728 – 62	Metal
M6x20-Bolt-DIN912	DIN 912 M6 x 20 stainless steel bolt	1	€ 0.21	€ 0.21	conrad.com 1205337 – 62	Metal
M6-Washer-DIN125	DIN 125 M6 stainless steel washer	16	€ 0.05	€ 0.88	conrad.com 1204371 – 62	Metal
M6-Washer-DIN9021	DIN 9021 M6 stainless steel washer	36	€ 0.20	€ 7.19	conrad.com 1204409 – 62	Metal
M5x20-Bolt-DIN912	DIN 912 M5 x 20 stainless steel bolt	4	€ 0.36	€ 1.44	conrad.com 1205330 – 62	Metal
M6x14-Bolt-DIN912	DIN 912 M6 x 14 steel zinc galvanized bolt	32	€ 0.15	€ 4.80	conrad.com 116756 – 62	Metal
bracket	Aluminium 90° Angle bracket 3030	16	€ 3.29	€ 52.64	conrad.com 2623302 – 62	Metal
profile	Aluminium profile 3030, 1000 mm	4	€ 20.99	€ 83.96	conrad.com 2623293 – 62	Metal
top_chamber	Top chamber (Estimated cost to print with 90.0 g of PLA-CF)	1	€ 3.57	€ 3.57	https://eu.store.bambulab.com/en-be/products/pla-cf	Polymer
working_fluid_chamber	Working fluid chamber (Estimated cost to print with 37.5 g of PLA-CF)	1	€ 1.49	€ 1.49	https://eu.store.bambulab.com/en-be/products/pla-cf	Polymer
electrolyte_chamber	Electrolyte chamber (Estimated cost to print with resin volume of 105 mL)	1	€ 28.26	€ 28,26	https://www.seido-solutions.com/en/ind403-high-modulus	Polymer
electrolyte_diaphragm	Deutsch Neuman 5,203,001 Fluoroelastomer diaphragm 300x300mm (Price calculated for a 100 mm x 100 mm square cut)	1	€ 8.33	€ 8.33	fishersci.fr 10536104	Polymer
working_fluid_diaphragm	ERIKS Rubber sheet EPDM 70 131304 10000x1400x3mm (Price calculated for a 100 mm x 100 mm square cut)	1	€ 1.21	€ 1.21	https://shop.eriks.nl/en/rubber-sheet-epdm-70-131304-10000x1400x3-10017116/	Polymer
straight tube connectors	emteknik 1C100MG4018PP Straight connector with connection for flexible tube (Series 1C) and male G-thread	4	€ 3.92	€ 15.68	https://em-technik.com/en/products/connecting/flexible-tube-connectors/connection-principle-1c/1c100mg-1/12121	Polymer
			**Total**	**€ 483.65**		

### 5.1 Build instructions

The overall device is composed of two main parts: (i) The aluminium support structure and (ii) the actual pulsator. An aluminium structure supports the pulsator, stepper motor, and rod guide. It is recommended to build the aluminium structural support and pulsator separately and then assemble them together.

### 5.2 Aluminium structural support building instructions

**Fig. 2:**
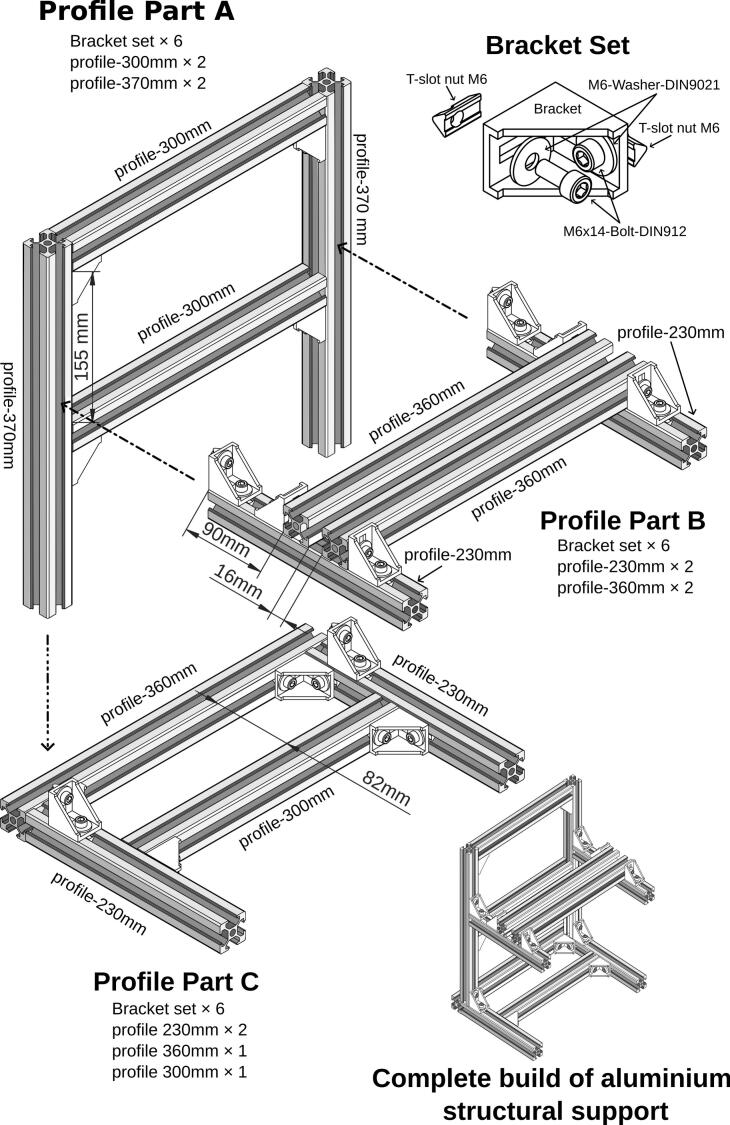
Building schematic and required parts for aluminium structural support components.

To construct the aluminium structural support, four 1-meter-long aluminium profiles were cut into twelve pieces, as specified in Table 1. For assembly, a set of 90° angle brackets, washers, nuts, and T-slot nuts is required. Detailed information on the necessary components for building the aluminium structural support profiles can be found in Fig. 2. For simplicity, the structure is divided into three sections—Part A, Part B, and Part C—which were first assembled separately and then combined. The aluminium profiles can be cut using either a hand saw or an electric saw. When assembling the bracket sets, it is preferable to use larger outer diameter M6-Washer-DIN9021 (18 mm diameter) instead of M6-Washer-DIN125 (12 mm diameter) to prevent bolt pull-through of the brackets. It is recommended to not over-tighten the bolts on the bracket sets that join Profile Part B to Profile Part A and Profile Part C to Profile Part A, as adjustments may be needed during the installation of the pulsator and stepper motor.

**Table 1:** Cutting plan for 1-meter aluminium profiles with kerf width assumption of 3  mm per cut.

Profile	Pieces Cut (mm)	Number of Cuts	Kerf Loss (mm)	Total Length Used (mm)	Waste (mm)
**1**	370, 370, 230	2	6	976	24
**2**	300, 300, 230	2	6	836	164
**3**	360, 360, 230	2	6	956	44
**4**	360, 300, 230	2	6	896	104

### 5.3 Motor Coupler and adjustable crank building instructions

The crank is designed to have an adjustable radius by turning an M6 × 80-Bolt-DIN933, which also holds the Motor Coupler and Adjustable Crank together. Motor Coupler, Adjustable Crank, rod guide, yoke, working_fluid_chamber, and top_chamber were 3D-printed using PLA-CF filament with a BambuLab Carbon X1E FDM printer, sliced using BambuStudio version 1.9.7.52. PLA-CF filament was chosen for its improved strength and stiffness over standard PLA filament.

**Fig. 3:**
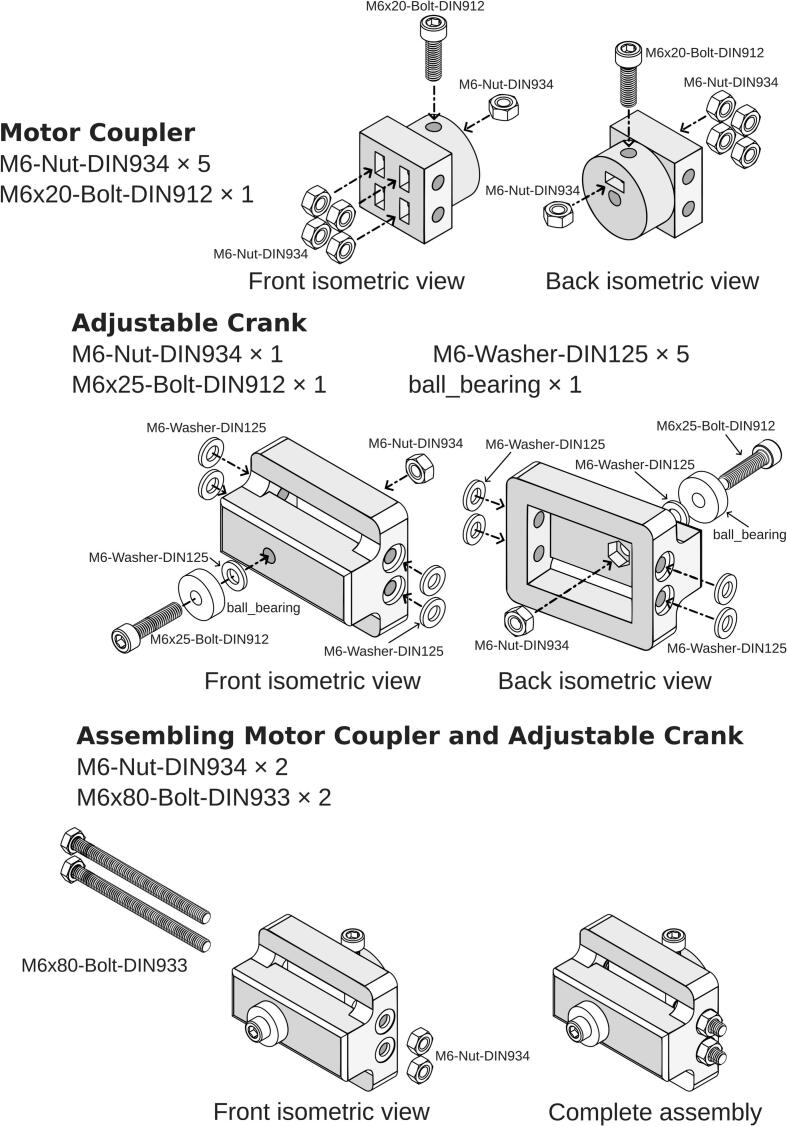
Assembly instructions for Motor Coupler and Adjustable Crank.

**Fig. 4:**
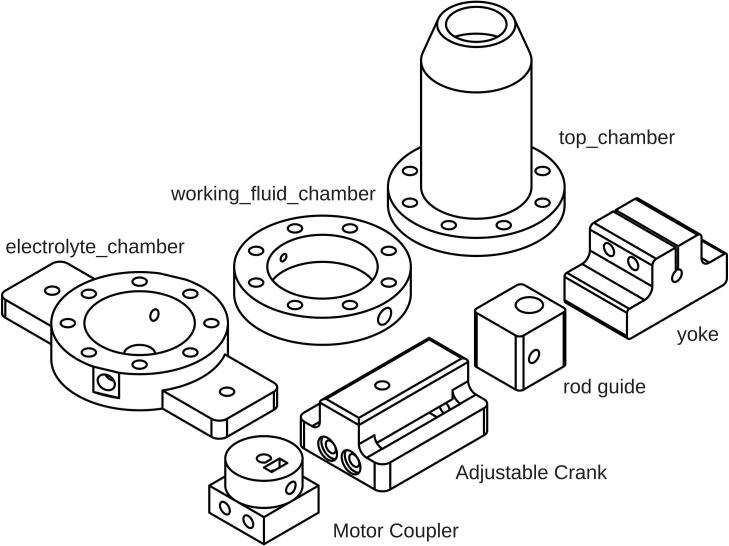
Print orientation for the 3D-printed parts using FDM. electrolyte_chamber are printed with SLA printer.

To ensure the strength of the Motor Coupler, the 3D printed wall loops were set to 20 layers, completely filling the infill volume, and the top and bottom shell layers were set to 5 layers each. For Adjustable Crank, rod guide, and yoke a gyroid infill pattern at 35 % density is used, with 5 wall loops and 5 layers for both the top and bottom shells. The 3D printer uses a 0.4 mm nozzle, and a layer height of 0.12 mm was chosen for optimal print quality. Detailed assembly instructions and the required parts are presented in Fig. 3. The print orientation of the parts is shown in Fig. 4. Support for overhang was needed for the Adjustable Crank, while other parts do not. The M6 × 20-Bolt-DIN912 on the Motor Coupler must be applied with thread locker and tightened after being inserted into the stepper motor. A thread locker is required, as the vibration during operation could loosen the bolt.

It is important that an M6 × 80-Bolt-DIN933 or a similar fully threaded bolt be used, because the four M6-Nuts-DIN934 on the Motor Coupler need to slide along the bolt when the crank radius is adjusted. The use of a partially threaded bolt would prevent the nuts from moving freely, limiting the adjustability of the crank. Finally, CRC Multi-Purpose Grease was applied to the ball bearing and the M6 × 80-Bolt-DIN933 to ensure smooth operation.

The radius of the crank can be adjusted between 0 mm and 20 mm, allowing for precise control over the output amplitude. The crank radius is reduced by turning the M6 × 80-Bolt-DIN933 bolts clockwise, while crank radius is increased by turning the bolts counterclockwise.

### 5.4 Pulsator build instructions

**Fig. 5:**
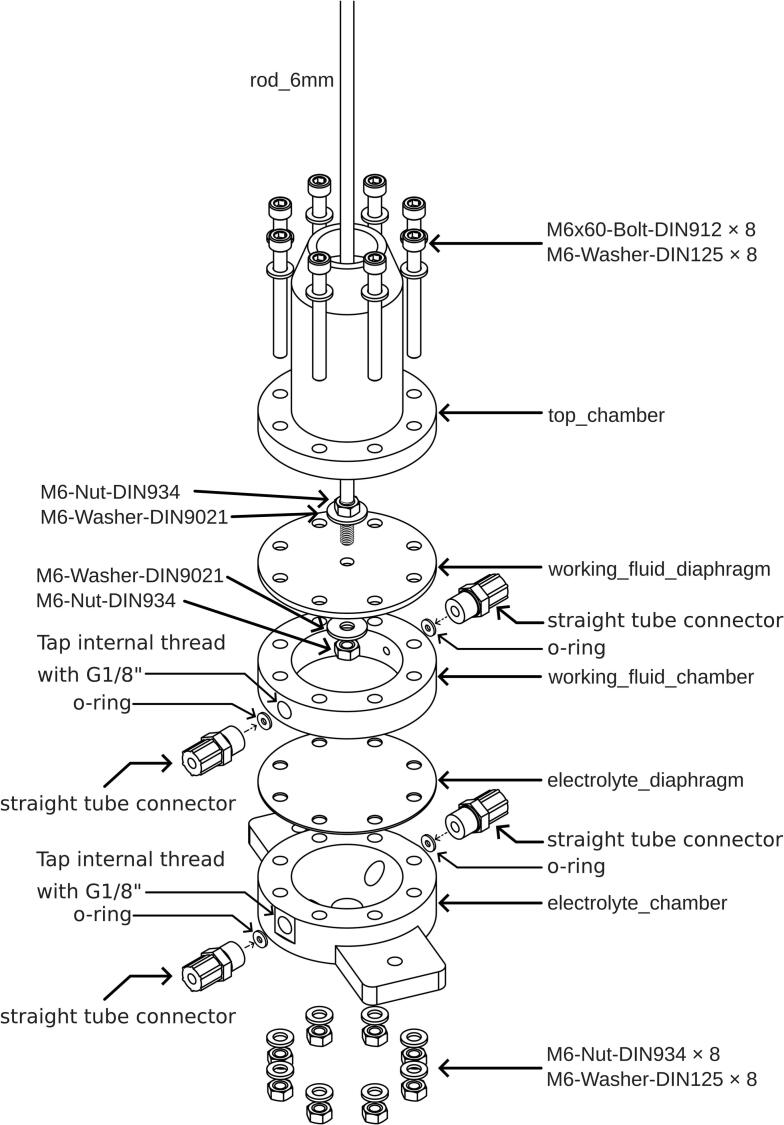
Assembly instructions for pulsator.

As mentioned above, the pulsator is divided into three compartments. The components that hold these chambers are respectively named electrolyte_chamber, working_fluid_chamber, and top_chamber. These chambers are separated by diaphragms; a 1 mm thick FKM diaphragm is placed between the electrolyte_chamber and the working_fluid_chamber, while a 3 mm thick EPDM diaphragm is placed between the working_fluid_chamber and the top_chamber. Fig. 5 shows the order of pulsator assembly and required components.

Both diaphragms were initially cut into circles with a diameter of 80 mm using a circle cutter, and eight 6 mm diameter holes were punched using a hole puncher. A hole in the centre is not present in the electrolyte_diaphragm, whereas a 6 mm hole in the centre is required for the working_fluid_diaphragm. To simplify the process of punching eight 6 mm diameter holes around the diaphragm, the working_fluid_chamber—which already has these holes incorporated during the 3D printing process—was used as a precise template. The chamber was placed on top of the diaphragm, ensuring proper alignment. Then, a pin or marker was used to transfer the positions of the existing holes from the chamber onto the diaphragm. By using the working_fluid_chamber as a template, accurate placement of the holes was ensured, and the punching process was made easier. The rod_6mm was cut into the length of 375 mm and is connected to the working_fluid_diaphragm using two sets of M6 washers (DIN9021) and M6 nuts (DIN934) as detailed in Fig. 5.

The top_chamber and working_fluid_chamber were fabricated using FDM 3D printing using the same 3D printer settings as Motor Coupler part, while the electrolyte_chamber was produced using stereolithography (SLA) on an Origin One 3D printer with Loctite IND403 resin. The print settings were configured to the default values for Loctite IND403 resin in conservative mode, with a layer resolution of 100 μm and a separation distance of 10 mm. The first layer exposure time was set to 23.342 s with an exposure delay of 130 s. For subsequent layers, the exposure time was 6.687 s with an exposure delay of 6 s. After printing, all SLA-printed parts were carefully removed from the build plate. The parts were cleaned using isopropanol in a Loctite EQ DW11 washing station. Two vessels of isopropanol were used, and the washing time was set to 5 min in each vessel at the high-speed setting. After cleaning, the parts were cured under UV light in the Loctite EQ CL36 LED Cure Chamber for 5 min at 100 % intensity. 3D-printed parts printed with FDM or SLA did not require any post-treatment. On the electrolyte_chamber and working_fluid_chamber, threads of size G1/8 were tapped into the two holes present on both chambers, intended for straight tube connectors (em-technik 1C100MG4018PP), which are designed for tubing sizes DN04/06. Before inserting the straight tube connectors into the printed parts, O-rings were fabricated by cutting an EPDM sheet into rings with an outer diameter of 8 mm and an inner diameter of 4 mm. These O-rings were placed inside the tapped holes, seated at the base of the threads, to ensure a proper seal when the connectors were installed.

When the pulsator is being assembled, it is important that the working_fluid_diaphragm be first attached to the rod_6mm. The nuts that hold the pulsator parts were tightened to 3 Nm using a torque wrench to ensure consistent compression and prevent leakage. If a torque wrench is unavailable, a standard wrench is acceptable if the applied torque prevents leakage without damaging the 3D-printed parts. Our tests show that 3D-printed parts can tolerate up to 5 Nm without significant damage. After assembly, it is recommended that a leak test be performed to verify the integrity of all seals and connections.

### 5.5 Installing pulsator and crank onto the aluminium structural support

**Fig. 6:**
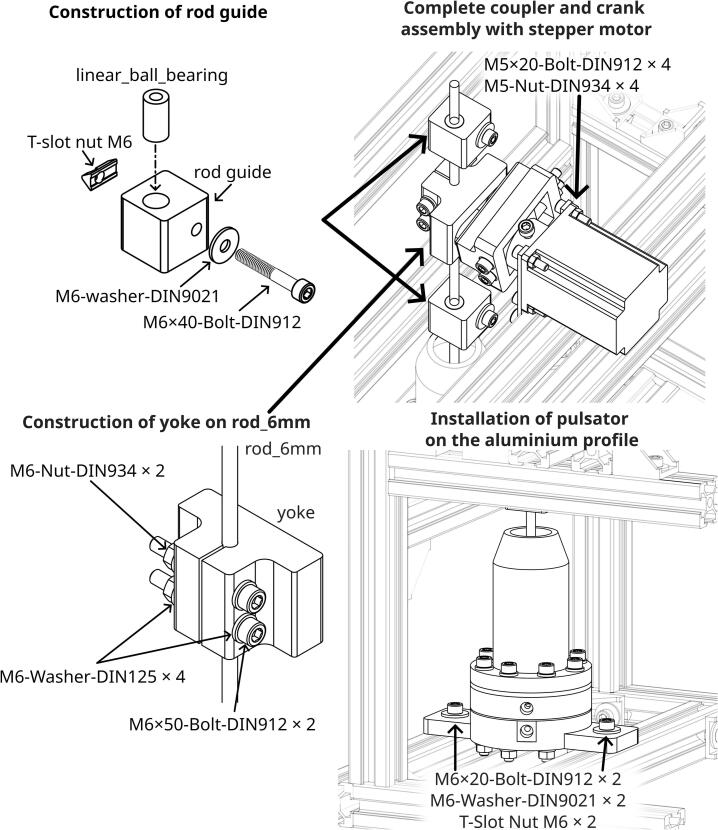
Build instructions and installation of rod guide, yoke, and pulsator on the aluminium structural support.

The required parts, along with detailed instructions for constructing the rod guides and installing the motor coupler, crank, and pulsator onto the aluminium structural support, are shown in Fig. 6. To construct a rod guide, a linear ball bearing is inserted into the rod guide piece. If the fit is too loose, tape should be wrapped around the linear ball bearing before insertion to achieve a snug fit. Two rod guides were needed in total. It is important that the yoke not be tightened onto the rod_6mm at this stage, as the position will need to be adjusted later. The bolts that hold the rod guides and pulsator should also remain loose until the rod_6mm is properly aligned and standing straight. It should be ensured that the rod moves smoothly through the rod guides without any binding or resistance. Friction was further reduced by the addition of lubricating grease on the linear_ball_bearing and inside the yoke. The NEMA-23–05 stepper motor was attached to the aluminium structural support profile using the aluminium_bracket_motor, which was milled from 10 mm thick aluminium plate using an imes-icore Euromod MP45 milling machine. Additionally, a 100 mm G-clamp can be used to secure the stepper motor to the aluminium structural support profile, helping to reduce vibration and noise. We utilised G-clamp because G-clamp design allows for quick attachment and detachment, simplifying adjustments and modifications during testing. It should be ensured that the centre of the motor shaft is horizontally aligned with the rod_6mm to prevent misalignment. The crank should not come into contact with the yoke to prevent excessive wear on the components.

**Fig. 7:**
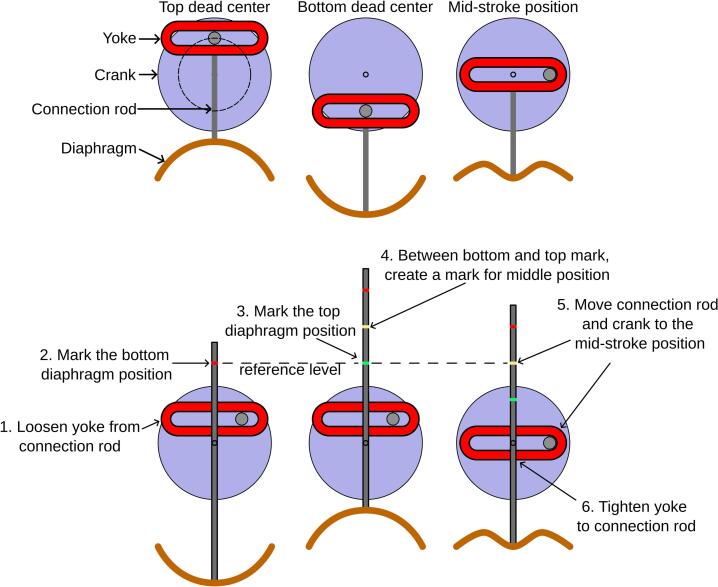
Instructions for positioning diaphragm and crank.

To ensure that the pulsator generates fluid pulses similar to a sine wave, it is critical that the crank be correctly positioned relative to the working_fluid_diaphragm. The crank's position must be adjusted to ensure that its rotational motion translates into the desired reciprocating motion of the diaphragm. The procedure for adjusting the yoke position is detailed in Fig. 7. Ideally, the distance between the yoke and the diaphragm should be such that when the crank is in the mid-stroke position, the diaphragm is also at its mid-stroke position.

Steps to adjust the yoke, connection rod, and crank position:

1. The yoke is loosened so that the connecting rod (rod_6mm) can slide freely.
2. The connecting rod is pushed down until the diaphragm rests at the bottom dead centre position.
3. The position of the connecting rod is marked at a chosen reference level (e.g., a fixed point on the structural support).
4. The connecting rod is pulled up until the diaphragm rests at the top dead centre position.
5. The position of the connecting rod is marked at the same reference level used in step 3.
6. The distance between the top and bottom marks on the connecting rod is measured. The midpoint of this distance is calculated, and a new mark is made at the centre between the two existing marks.
7. The connecting rod is adjusted so that the middle position mark aligns with the reference level. This positions the diaphragm at its mid-stroke point.
8. The crank is rotated to mid-stroke rotation.
9. The yoke is securely tightened onto the connecting rod at this position, ensuring it does not shift.

### 5.6 Stepper motor control with Arduino: Wiring instructions

**Fig. 8:**
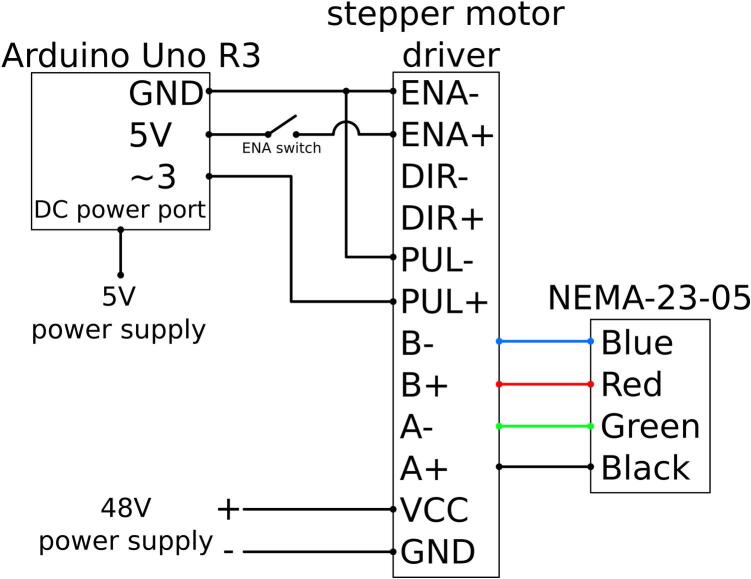
Wiring schematic between Arduino, stepper motor driver, and stepper motor.

Electrical pulses are generated by the Arduino Uno R3 for the stepper motor driver, which is then used to control the NEMA-23–05 stepper motor. Optimal motor performance is achieved through proper configuration of the TB6600 stepper motor driver and careful adjustment of settings. The electrical wiring is shown in Fig. 8.

Stepper Motor Driver Current Settings:

• Initial Current Setting: The DIP switches on the TB6600 driver should be set to provide an initial current of 0.5 A.
• Adjusting Current: If the motor operates without stalling, the current may gradually be increased as needed, based on the load requirements.
• Caution: More current than necessary should not be supplied. Excessive current can cause the NEMA-23–05 stepper motor to vibrate, stall, or overheat. Power should be disconnected before making wiring changes.

Pulse per Revolution (rev) Settings:

• DIP Switch Configuration: The pulse/rev (microstepping) setting on the driver should be set to 200 pulses/rev. Microstepping can be increased to achieve smoother motor rotation, provided that the motor does not stall.
• Performance Note: Setting the pulse/rev higher than 400 pulses/rev may cause the motor to stall at speeds above 120 revolutions per minute (RPM) due to increased step frequency.
• Caution: The stepper motor should be turned off before servicing. Hands and fingers should be kept clear from the stepper motor while the pulsator is in operation.

### 5.7 Filling working fluid instructions

**Fig. 9:**
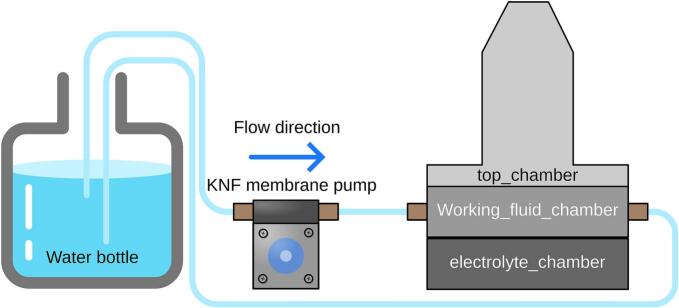
Schematic for filling working_fluid_chamber with water.

To fill the working_fluid_chamber with the working fluid and ensure all air bubbles were removed, sections of flexible PVC tubing, each 50 cm in length, were connected to the straight connectors of the working_fluid_chamber. The inlet of the chamber was connected to a KNF NF1.25RTDCB-4B membrane pump (hereafter referred to as the KNF membrane pump), which was used to draw water from a reservoir bottle. The outlet of the chamber was directed back into the reservoir, creating a closed-loop system. A relevant schematic is shown in Fig. 9.

To facilitate the removal of air bubbles from the working_fluid_chamber, the pulsator was turned on. The crank radius was set to 15 mm, and the program increase_speed_step_loop.ino was uploaded to the Arduino. The stepper motor driver was configured with a stepping resolution of 200 steps/rev and a current setting of 0.5 A. Both the KNF membrane pump and the pulsator were operated simultaneously until no air bubbles were observed within the system.

After purging the system of air, tubing clamps were placed on the PVC tubing near the straight connectors to seal off the working_fluid_chamber. The KNF NF1.25RTDCB-4B membrane pump was utilized due to its availability at the time of the experiment; however, if this pump was not available, any pump with a flow rate exceeding 50 mL/min can be used as a replacement.

The top_chamber was also filled with working fluid to prevent spillage from the working_fluid_chamber and to inhibit air from entering the system. By partially filling the top_chamber with water (filling it halfway is sufficient), air ingress into the working_fluid_chamber was effectively prevented.

**Fig. 10:**
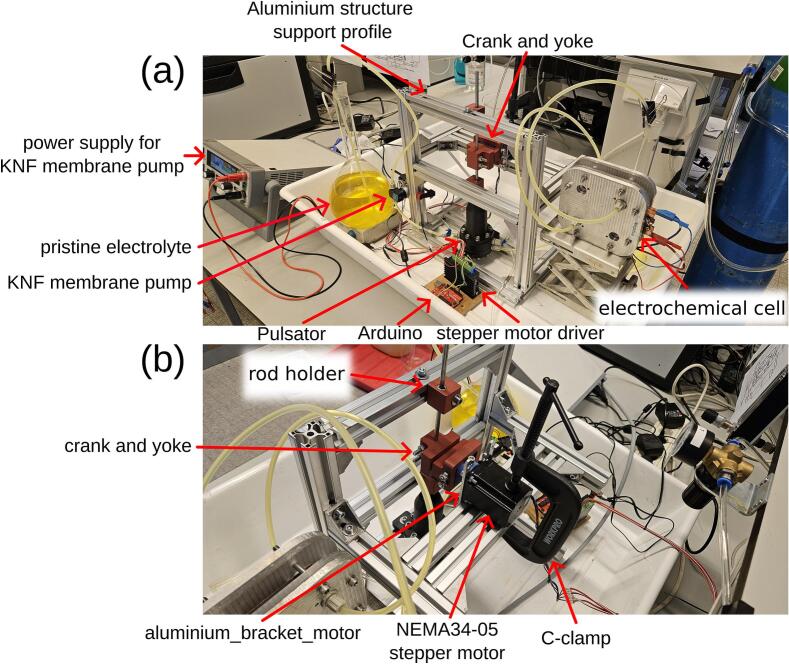
(a) Photograph of the diaphragm pulsator integrated with the electrochemical cell used for the limiting current experiment. (b) Close-up photograph of the crank-yoke mechanism connected to the stepper motor.

A photograph of a complete assembly of the pulsator on the aluminium structural support profile is shown in Fig. 10.

## 6 Operation instructions

1. The Arduino IDE is opened, and the desired program is uploaded to the Arduino board.
2. The crank diameter is set to the desired length according to experimental requirements.
3. The USB cable between the Arduino and the computer is disconnected.
4. A 5 V power supply is connected to the Arduino.
5. The ENA switch on the stepper motor driver is set to the ON position to turn off pulses to the stepper motor. Note: The ENA switch controls the enable function of the TB6600 stepper motor driver. When the ENA switch is turned ON, the motor is disabled without cutting power to the driver.
6. A 48 V power supply is connected to the stepper motor driver.
7. The microstepping settings on the stepper motor driver are configured according to the desired experimental requirements.
8. Turn on the pulsator:
  ◦ The Arduino is reset by pressing the reset button.
  ◦ Immediately, the ENA switch on the stepper motor driver is turned to the OFF position to activate the pulsator.
9. Turn off the pulsator:
  ◦ The ENA switch is turned back to the ON position on the stepper motor driver to deactivate the pulsator.

## 7 Validation and characterization

### 7.1 Validation of pulsator flow profile

**Fig. 11:**
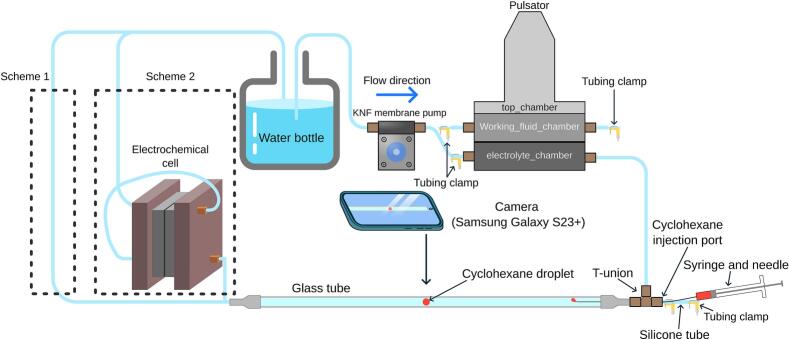
Schematic for the flow profile experiment.

To verify whether the flow generated by the diaphragm pulsator resembles a sinusoidal wave, a camera tracking setup was established, as shown in Fig. 11. Cyclohexane (VWR Chemicals BDH) was injected into a 1 m long glass tube with an inner diameter of 8 mm to create a droplet visible to the camera. When the pulsator was activated, the cyclohexane droplet was observed to oscillate in response to the motion of the liquid.

Water was selected as the working fluid for the study, while a cyclohexane droplet was chosen as the tracking particle due to its immiscibility with water, low viscosity, and incompressibility compared to air bubbles. Red oil-soluble dye was added to the cyclohexane to enhance visibility during video tracking. The video analysis software Tracker version 6.2.0 was utilized to monitor the movement of the cyclohexane droplet, operating under the assumption that the droplet's motion represents the flow profile generated by the pulsator, which was observed to have a nearly negligible drift during the timescale of the measurements.

To prepare the glass tube for the experiment, the glass tube was treated overnight with a diluted solution of RBS 25 concentrate (Chemical Products R. Borghgraef S.A.) at a concentration of 20 mL/L. This treatment of the inner wall of the glass tube prevented the cyclohexane droplet from adhering to the wall, ensuring smooth droplet motion. The internal volume of the glass tube was determined by filling it with 60 mL of water and recording the corresponding height. For a travel distance of 30.0 cm, a displacement of 14.57 mL of water was observed. Volume calibration markers were placed on the glass tube so that they were visible to the camera, facilitating accurate volume measurements during video tracking. A slow-motion video at 240 fps was captured using a Samsung Galaxy S23 + in slow-motion mode. The camera was mounted on a stand and positioned 50 cm above the glass tube.

Before recording the flow profile, air bubbles inside the pulsator and glass tube were removed by turning on the membrane pump at a flow rate of 200 mL/min. The pulsator was also turned on to facilitate the removal of air bubbles. Flushing continued for 10 min until no more air bubbles were observed.

A cyclohexane droplet of approximately 0.5 mL was injected through the silicone tube at the injection port, creating a cyclohexane droplet of 8 mm in diameter. To prevent water leakage from the silicone tube, a tubing clamp was placed between the injection hole and the T-union. The membrane pump was turned on until the cyclohexane droplet travelled to a location within the glass tube where it was visible to the camera. Before conducting any flow profile experiments, the tubing clamp between the membrane pump and pulsator was tightened to prevent backflow into the membrane pump.

**Fig. 12:**
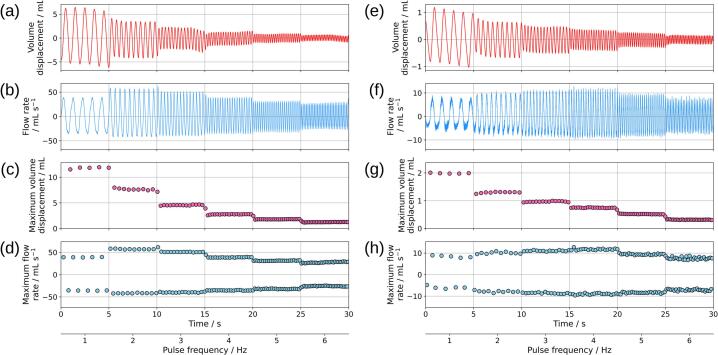
Flow profiles generated by the diaphragm pulsator using a 15 mm diameter crank. Panels (a)–(d) represent the system without the electrochemical cell connected, while panels (e)–(h) show the system with the electrochemical cell connected. Specifically, (a) and (e) display the volume displacement over time, and (b) and (f) illustrate the flow rate over time. Panels (c) and (g) highlight the maximum volume displacement, and panels (d) and (h) present the maximum flow rate observed. Negative flow rates indicate reverse flow direction.

The results of the video tracking analysis, with corrections for cyclohexane droplet drift are presented in Fig. 12. In our analysis, “drift” refers to the net displacement of the cyclohexane droplet that occurs even in the absence of a net flow generated from a pump. Ideally, the cyclohexane droplet will rest at the same location after a complete cycle of pulsation. However, this is observed not to be the case; we propose a hypothesis that during pulsation, liquid flow occurs through the thin film between the cyclohexane droplet and the glass tube. The volume of liquid flowing through the thin film is not equal between the forward pulse and the backward pulse, which resulted in a net flow.

To isolate the pulsatile movement, we used a locally weighted scatterplot smoothing (LOWESS) algorithm via the statsmodels Python library to extract and subtract the drift component from the raw cyclohexane droplet positional data. By adjusting the fraction parameter in LOWESS, we extract the underlying drift pattern. The drift component is then subtracted from the raw positional data obtained via video tracking. The Python scripts used for the analysis are included in the same repository as the CAD file. The stepper motor program used, increase_speed_step.ino, was configured with a microstepping setting of 200 steps/rev and a current of 1.0 A. The crank rotation was initiated by this program at 1 Hz (60 RPM), increasing by 1 Hz every 5 s up to a maximum of 6 Hz (360 RPM).

**Fig. 13:**
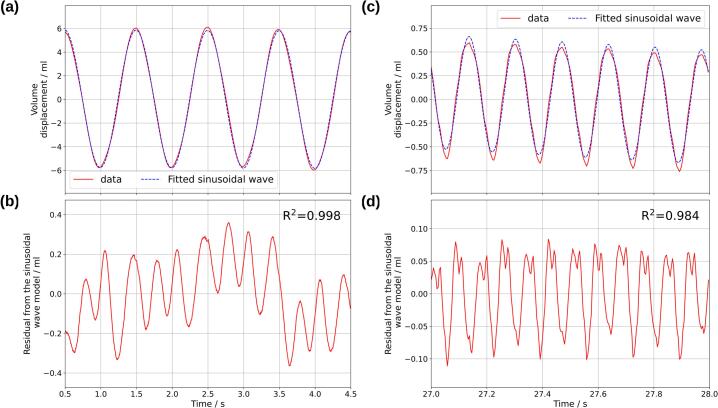
Volume displacement flow profiles of the pulsator with a 15 mm diameter crank and without the electrochemical cell connected. Subplots (a) and (b) correspond to a pulse frequency setting of 1 Hz, while subplots (c) and (d) correspond to a pulse frequency setting of 6 Hz. In subplots (a) and (c), sinusoidal wave fittings are applied to the flow profiles generated by the pulsator. Subplots (b) and (d) present the residuals and coefficients of determination (R^2^ values) for each sinusoidal fit. The fitting parameters for subplot (a) include a peak-to-peak amplitude of 11.63 mL, frequency of 1.00 Hz, phase shift of −1.5 rad, offset of 0.08 mL, and a slope of −0.03 mL/s. The fitting parameters for subplot (a) include a peak-to-peak amplitude of 1.20 mL, frequency of 5.99 Hz, phase shift of 4.86 rad, offset of 4.59 mL, and a slope of −0.17 mL/s.

Panels (a)–(d) in Fig. 12 illustrate the system without the electrochemical cell connected (scheme 1), while panels (e)–(h) show the system with the cell connected (scheme 2). In both cases, the flow profiles displayed in panels (a) and (e) exhibit sinusoidal characteristics, as confirmed by the sinusoidal wave fittings applied in Fig. 13, with drift correction applied. The fitting equation for the sinusoidal curve is given in Equation (1), where *y* represent volume displacement (mL); *A* represents amplitude (mL), with peak-to-peak amplitude equal to *2 × A*; *f* is the frequency (Hz); *t* is time (s); *θ* is the phase shift (radians); *m* is the slope (mL/s); and *b* is the offset (mL). The term *mx + b* is a linear fitting that corrected for drift. Relevant parameters for discussion include the peak-to-peak amplitude and frequency. Coefficient of determination (R^2^) was calculated using Equation (2) where $y_{i}$ is the observed value, $f_{i}$ is the fitted value, and $\overset{¯}{y}$ is the mean of the observed value.

(1) $$y=Asin\left(2\pi ft+\theta \right)+mt+b$$

(2) $$R^{2}=1-\frac{\sum_{i}{\left(y_{i}-f_{i}\right)}^{2}}{\sum_{i}{\left(y_{i}-\overset{¯}{y}\right)}^{2}}$$

**Fig. 14:**
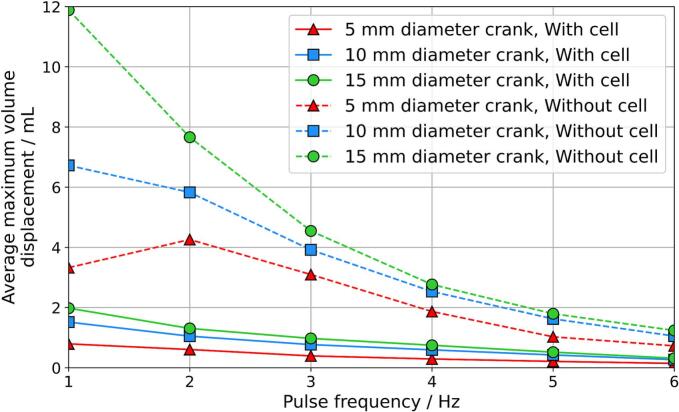
Average maximum volume displacement at pulse frequencies from 1 to 6 Hz, with and without the electrochemical cell connected.

Fig. 13 demonstrates that the flow profile aligns closely with a sinusoidal wave, with R^2^ values for the volume displacement profile at 15 mm crank diameter showing 0.998 at 1 Hz and 0.984 at 6 Hz. The volume displacement over time was differentiated to obtain the flow rate over time, shown in Fig. 12 (b) and (f), where negative flow rates indicate reverse flow. Panels (c) and (g) in Fig. 12 present the maximum peak-to-peak volume displacement, and panels (d) and (h) show the maximum peak-to-peak flow rate. In Fig. 12 (c) and (g), a trend of decreasing peak-to-peak amplitude of volume displacement was observed as pulse frequency increases from 1 Hz to 6 Hz. The maximum volume displacement was 11.9 mL and was reduced to 2.0 mL with the electrochemical cell connected. However, the flow rate peaks at 2 Hz in Fig. 12 (d), reaching a maximum of 57.0 mL/min for forward flow and 41.5 mL/min for backward flow. When the electrochemical cell was connected, as shown in Fig. 12 (h), the maximum forward and backward flow rates occur at 4 Hz, with values of 11.7 mL/min and 8.9 mL/min, respectively. A summary of the flow profiles for crank diameters of 5 mm, 10 mm, and 15 mm is provided in Fig. 14. It should be noted that the electrochemical cell was inactive during these flow profile experiments and was only activated during the limiting current experiments. The flow profile was unaffected by the status of the electrochemical cell (i.e., on–off).

### 7.2 Validation of mass transfer enhancement in an electrochemical flow cell

**Fig. 15:**
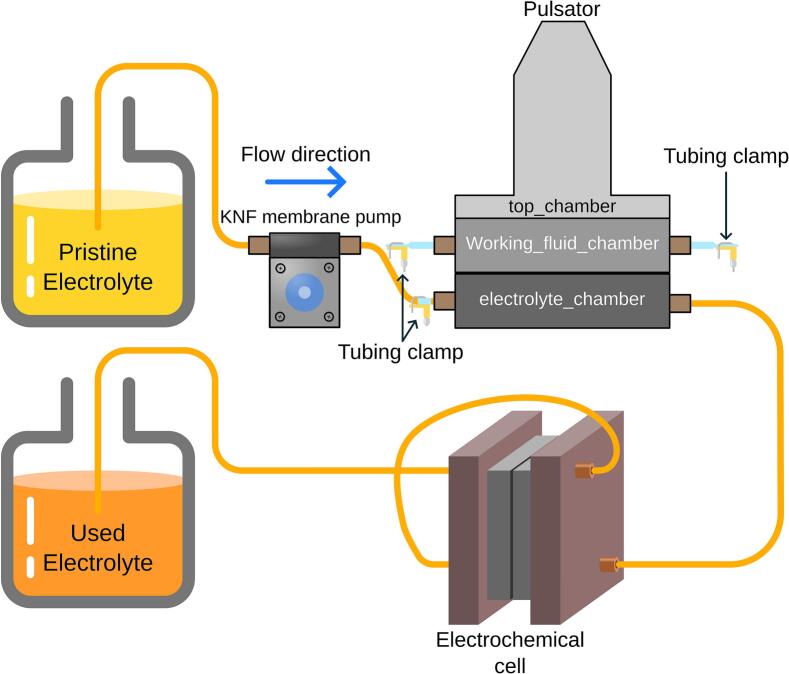
Limiting current experiment of ferri/ferro cyanide with single pass configuration.

To investigate the effects of pulsation on a laboratory-scale electrochemical reactor, a ferri/ferrocyanide electrolyte in potassium carbonate was used in a limiting current experiment [17], [18]. The electrochemical cell was connected to the diaphragm pulsator, as illustrated in Fig. 15. A photograph of the fully assembled diaphragm pulsator integrated with the electrochemical cell setup is shown in Fig. 10. A total of 4 L of electrolyte was required, prepared in two separate 2 L batches. The electrolyte concentration was set at 1 M K_2_CO_3_, 25 mM ferricyanide, and 100 mM ferrocyanide. The rate-limiting reaction was ensured to depend on ferricyanide by the excess ferrocyanide [17], [18]. The frequency of the pulsator was set to 1 Hz and a crank diameter of 15 mm (volume displacement of 2.0 mL).

To prepare 2 L of electrolyte solution, 276.41 g of ≥ 99 % anhydrous potassium carbonate (K_2_CO_3_, Chem-Lab NV), 84.48 g of 98.5 % potassium ferrocyanide trihydrate (Thermo Fisher Scientific), and 16.46 g of ≥ 99 % potassium ferricyanide (VWR Chemicals BDH) were added to a 2 L volumetric flask, and Milli-Q water was added to bring the solution to the 2 L mark.

The electrochemical cell consists of a graphite rectangular channel with each cell compartment measuring 4.00 cm in width, 5.00 cm in length, and 3.58 mm in depth, resulting in a projected electrode surface area of 20 cm^2^. Two 4 cm × 5 cm polypropylene mesh pieces, each with a porosity of 0.72, were used as turbulence promoters in each cell compartment [19]. The two compartments were separated by a Nafion 117 cation exchange membrane. The electrolyte flow was configured as a divided cell, on the cathode side, the ferricyanide was reduced to ferrocyanide according to Equation (3).

(3) $${\left[Fe\left({\mathrm{CN}}_{6}\right)\right]}^{3-}+e^{-}\to {\left[Fe\left({\mathrm{CN}}_{6}\right)\right]}^{4-}$$

Under mass transport control conditions, the mass transport coefficient (*k*) is related to the limiting current (*I_L_*) as described by Equation (4) where *k* is the mass transport coefficient (cm/s), *I_L_* is the limiting current (A), *A_e_* is the electrode area (cm^2^), *z* is the number of electron transfers, *F* is Faraday's constant (A·s/mol), and *c_∞_* is the bulk concentration of ferricyanide in the electrolyte (mol/L) [18].

(4) $$k=\frac{I_{L}}{A_{e}zFc_{\infty }}$$

The mean linear flow velocity (*v*) in the cell compartment was calculated using Equation (5) where *v* is the mean linear flow velocity (cm/s), *Q* is the flow rate (mL/s), *W* is the width of the cell compartment (cm), *h* is the depth of the cell compartment (cm), and *ϕ* is the porosity of the cell compartment.

(5) $$v=\frac{Q}{Wh\phi }$$

To compare the performance of pulsation to constant flow, the mass transport enhancement factor (γ) is introduced and defined as shown in Equation (6) where $k_{\mathrm{pulsating}}$ is mass transport coefficient with pulsator turned on and $k_{\mathrm{continuous}}$ is mass transport coefficient with only constant flow [17].

(6) $$\gamma =\frac{k_{\mathrm{pulsating}}}{k_{\mathrm{continuous}}}$$

**Fig. 16:**
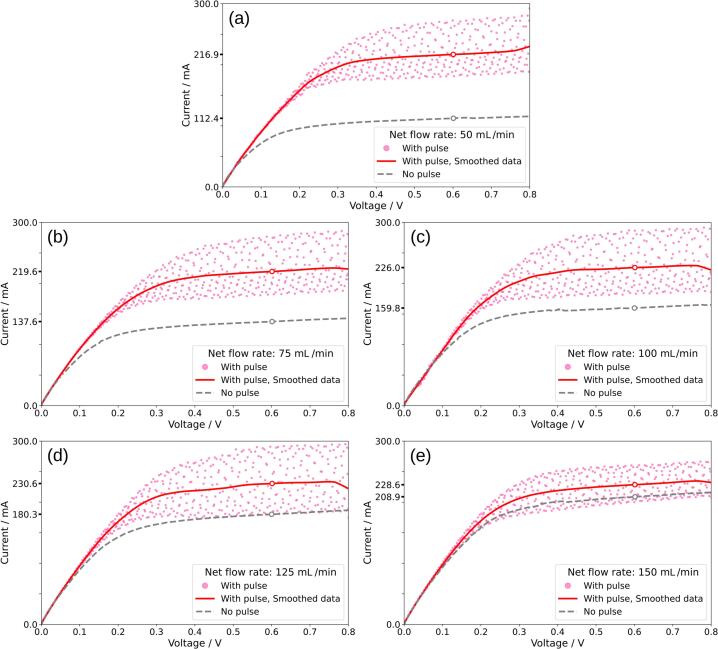
Limiting current measurements for the ferro/ferricyanide redox couple, obtained with the pulsator set to a crank diameter of 15 mm and a pulse frequency of 1 Hz. Subplots (a), (b), (c), (d), and (e) correspond to net electrolyte flow rates of 50, 75, 100, 125, and 150 mL/min, respectively.

**Fig. 17:**
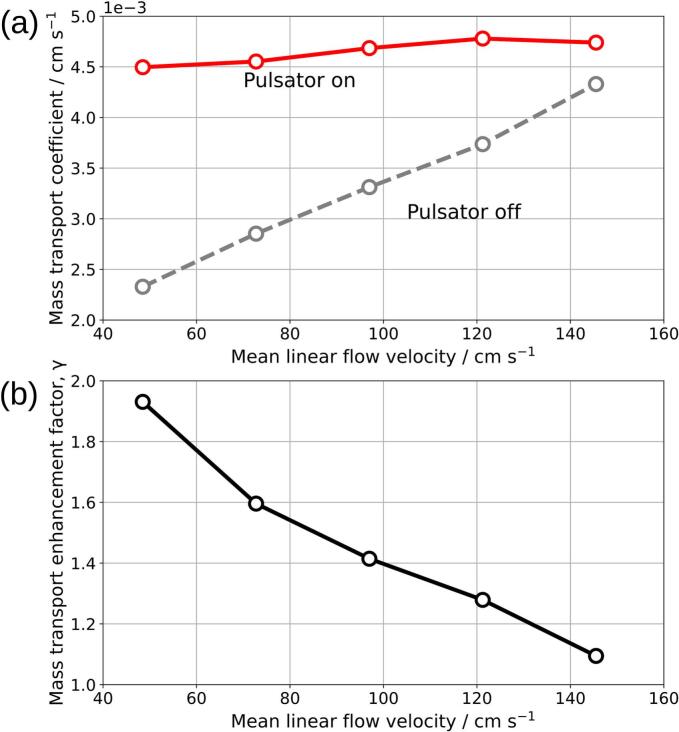
(a) Mass transport coefficient of flat electrode vs. mean linear flow velocity of an electrolyte in the flat electrode with and without pulsation. (b) Mass transport enhancement factor as a function of mean linear flow velocity.

A Biologic BCS-815 battery cycler was used to perform linear sweep voltammetry (LSV) to determine the limiting current, employing a sweep rate of 5 mV/s over a potential range from 0 V to 0.8 V. The limiting current was measured at 0.6 V, where the current reached a plateau, indicating a reaction operating within the mass transport-controlled region. Fig. 16 (a), (b), (c), (d), and (e) show the limiting current experiment results for net electrolyte flow rates of 50, 75, 100, 125, and 150 mL/min, respectively, while Fig. 17 (a) shows the calculated mass transport coefficient plotted against the mean linear flow velocity. The measured current for pulsating flow in Fig. 16 was smoothed with LOWESS to obtain the average current value. The mass transport coefficient for the pulsating electrolyte flow ranged from 4.50 × 10^−3^ cm/s to 4.78 × 10^−3^ cm/s at mean linear velocities between 48.5 cm/s and 145 cm/s, respectively, whereas for the constant flow rate the mass transport coefficient ranged from 2.33 × 10^−3^ cm/s to 4.33 × 10^−3^ cm/s. Fig. 17 (b) illustrates γ for the flow cell with a turbulence promoter. The values decrease from 1.93 to 1.09 for the mean linear velocities of 48.5 cm/s and 145 cm/s, respectively. To put this in perspective, the mass transport enhancement factor of the turbulence promoter alone in respect to a planar electrode can be up to ≈4 under continuous flow [17]. The limiting current experiments conducted in this study show that the implementation of pulsating flow, generated by the diaphragm pulsator at 1 Hz and volume displacement of 2.0 mL enhances mass transfer for the ferri/ferrocyanide reaction in the evaluated electrochemical flow cell compared to constant flow conditions, particularly at low flow rates. These results agree with previous work, where performance improvements were observed in organic electrosynthesis reactors [3] and flow batteries [7] when they operated in mass transport limited conditions under pulsating flow. In further studies, electrolyte pulsation could be combined with additional mass transport enhancing methods such as other types of turbulence promoters [20], [21], ultrasound [22] and well-known 3D porous electrodes [23].

### CRediT authorship contribution statement

**Kavin Teenakul:** Writing – original draft, Visualization, Methodology, Investigation, Conceptualization. **Luis Fernando Arenas:** Writing – review & editing, Supervision, Methodology. **Jonas Hereijgers:** Writing – review & editing, Supervision, Funding acquisition, Conceptualization.

## Declaration of competing interest

The authors declare that they have no known competing financial interests or personal relationships that could have appeared to influence the work reported in this paper.
